# Patients with chronic Achilles tendon rupture have persistent limitations in patient-reported function and calf muscle function one year after surgical treatment – a case series

**DOI:** 10.1186/s40634-022-00451-5

**Published:** 2022-02-09

**Authors:** Anna Nordenholm, Niklas Nilsson, Eric Hamrin Senorski, Katarina Nilsson Helander, Olof Westin, Nicklas Olsson

**Affiliations:** 1 Department of Health and Rehabilitation, Institute of Neuroscience and Physiology, Sahlgrenska Academy, University of Gothenburg, Gothenburg, Sweden; 2 Sportrehab Sports Medicine Clinic, Gothenburg, Sweden; 3 Department of Orthopaedics, Institute of Clinical Sciences, Sahlgrenska Academy, University of Gothenburg, Göteborgsvägen 31, 431 80 Mölndal Gothenburg, Sweden

**Keywords:** Chronic Achilles tendon rupture, Surgical treatment, Outcome, Achilles tendon rupture score, Calf muscle endurance, Tendon length

## Abstract

**Purpose:**

Evaluate the one-year postoperative outcomes in patients with Chronic Achilles tendon rupture.

**Methods:**

Patients surgically treated for Chronic Achilles tendon rupture (*n* = 22, 14 males and 8 females, mean age 61 ± 15) were evaluated by Achilles tendon Total Rupture Score, The Physical Activity Scale, The Foot and Ankle Outcome Score, Calf muscle endurance test, counter movement jump, Hopping, ultrasound measurement of tendon length, Achilles Tendon Resting Angle, dorsi flexion range of motion and calf muscle circumference. Muscle function and tendon length outcomes on the injured side were compared with the healthy side.

**Results:**

The patients scored a mean of 62 ± 26 on the Achilles tendon Total Rupture Score. Median scores on the injured compared with the healthy side were lower in heel-rise repetitions (20 vs 24 cm, *p* = 0.004), hel-rise height (8 vs 10 cm, *p* < 0.001), heel-rise total work (872 vs 1590 joule, *p* < 0.001) and hopping ratio (0.37 vs 0.48, *p* = 0.005). Median calf circumference was smaller (37 vs 38 cm, *p* = 0.001) and the mean tendon elongation greater on the injured side; Achilles tendon resting angle (55 vs 50°, *p* < 0.001) and ultrasound (22.4 vs 20.5 cm, *p* = 0.006).

**Conclusions:**

At one year postoperatively, patients with chronic Achilles tendon rupture reported persistent limitations in subjective foot and ankle function. Heel-rise height and total work as well as hopping ratio were not recovered, and there was an elongation of the injured Achilles tendon compared with the healthy tendon.

**Level of evidence:**

IV

## Introduction

The majority of Achilles tendon ruptures (ATR) occur during intense sporting activities and are most common in middle-aged men [8, 10, 14]. However, ATR has become more common in individuals over 60 years of age, in whom the injury mechanism is usually associated with less force and previous Achilles tendinopathy [8–10, 17, 18]. The overall incidence of ATR has been reported to range between 8 to 29 per 100,000 person-years [10, 16, 22, 35, 40]. Clinical examination and medical history are usually enough to diagnose an acute ATR [20, 23, 27, 33]. However, as many as 25% of the acute ATRs are reported to be missed in the acute phase, either due to a misdiagnosis by the assessing physician or because of patient delay [9, 24, 25]. An atypical patient history and clinical presentation makes the assessment more difficult and may result in the ATR being mistaken for another diagnosis, typically a tendinopathy, ankle sprain or calf muscle tear [2, 9]. If the rupture occurs spontaneously during daily activities or in conjunction with minor trauma, the patient may not understand the extent of the injury and may therefore wait to seek treatment as swelling and probably pain decrease [21].

In the absence of treatment for more than four weeks, the tendon ends retract and the gap is filled with fibrous tissue, resulting in the rupture being defined as a “chronic” Achilles tendon rupture (CATR) [21, 25]. Patients will eventually seek medical care complaining of pain and functional deficits such as weakness at push-off and poor balance, which creates significant alterations in the gait pattern [4, 12, 25]. Surgical repair is the treatment of choice for improving muscular function in the lower leg in patients with CATR [21, 25]. The surgical repair of CATR is more difficult than the surgical repair of an acute ATR (AATR) due to the retraction of tendon stumps and scar tissue formation; techniques beyond end-to-end repair are therefore required [4, 25].

Despite the fact that CATR occurs in up to one in four patients with ATR, there is currently limited knowledge with regard to the outcomes after surgical treatment [1, 13, 25]. Previous literature reports that impairments in ankle-related function may persist for several years after surgical treatment of CATR, but there is a widespread use of non-validated outcome measures [1, 3]. The aim of this study was to evaluate the one-year postoperative outcomes in patients with CATR using a comprehensive battery including several validated tests.

## Material and methods

The inclusion criteria were patients with a unilateral mid-portion CATR, defined as a mid-portion ATR that had been left untreated for at least four weeks. Patients with CATR that were surgically treated at Sahlgrenska University Hospital or Kungsbacka Hospital between 2014 and 2016 were invited to participate. A total of 22 patients (of which five patients were recruited at Kungsbacka Hospital) met the inclusion criteria and agreed to participate. All the patients received surgical treatment between one and 36 months after injury (mean 9, median 7 months) and were evaluated using the same standardized method at one year postoperatively. All the patients gave their written informed consent to take part in the study. Ethical approval was obtained from the Regional Ethical Review Board in Gothenburg, Sweden (DNR 554–15).

### Surgical treatment and postoperative care

Twenty-one of the 22 patients with CATR were treated with augmentation with a free flap from the gastrocnemius aponeurosis, a surgical technique previously presented by Nilsson Helander et al. [29]. One of the patients also received a suture anchor in the calcaneus due to a distal but still mid-portion location of the rupture. In one patient, a free semitendinosus autograft was used instead, because of a larger tendon gap. Postoperatively, a below-the-knee plaster cast was used for three to five weeks, followed by a adjustable lower-leg brace (DonJoy ROM Walker) for four to six weeks, which was removed after a total of 8–9 weeks after surgery. Toe-touch gait was allowed from three weeks and was successively increased to full weight-bearing in the brace, which was allowed within six weeks. The patients were referred to physical therapy for exercise therapy and load instructions at eight weeks postoperative.

### Follow-up

The follow-up of patients with CATR took place one year after surgery and was led by an experienced physical therapist. The evaluations were performed at the same orthopedic laboratory, and with the same measurements and tests, as in the study by Olsson et al. [32]. The tests were, however, led by a different physical therapist [32]. The follow-up consisted of a battery of tests including several validated tests for patient-reported outcomes, muscle function and clinical measurements of tendon length. Except from superficial wound complications in one patient, no complications were reported.

#### Patient-reported outcome measurements

##### The Achilles tendon total rupture score

The Achilles tendon total rupture score (ATRS) was the primary patient reported outcome measure and is an injury-specific instrument with high reliability, validity and sensibility for measuring outcome after ATR with high internal consistency (ICC = 0.96) and intraclass correlation (ICC = 0.98) [30]. ATRS contains 10 questions with symptom scales ranging from 0 to 10, resulting in a sum of 100, which means the patient has recovered completely. A lower score indicates more symptoms and greater limitations in function.

##### The physical activity scale

The physical activity scale (PAS) evaluates the regularity and level of activity among patients [11]. The scale consists of six levels of activity where the first level means very little activity and the sixth level means intense workouts numerous days a week.

##### The foot and ankle outcome score

The Foot and Ankle Outcome Score (FAOS) is valid and reliable for evaluation of patient outcome related to ankle reconstruction, but is commonly used also in patients with ATR [34]. The score measures patient outcomes in five sub-scales; Symptoms and stiffness, Pain, Function in daily living, Sport and recreation and Quality of life. The total score ranges from 0 to 100, with 100 indicating full recovery and no foot- and ankle-related problems, whereas a score of 0 indicates severe problems [34].

#### Muscle function tests

All the patients were given standardized instructions of the test procedure and warm-up. They wore standardized athletic footwear and were given verbal encouragement during the tests. For the evaluation of calf muscle endurance and jumping performance, the MuscleLab® (Ergotest Technology, Oslo, Norway) measurement system was used.

##### Calf muscle endurance

A single-leg standing heel-rise was used to evaluate calf muscle endurance. This test is widely used and is reliable and valid for patients with ATR [37]. Standing on a box with a 10-degree incline and allowed to use balance support with the fingertips on the wall at shoulder height, the patients were told to go as high as possible during each heel-rise, while keeping the knee extended. A metronome was used to keep the pace at 30 heel-rises a minute and the test was finished when the patients were unable to rise above 2 cm or were unable to maintain the pace. The linear encoder unit connected to the MuscleLab® measurement system recorded the number of successful heel-rise repetitions, the mean height of the heel-rise repetitions (cm) and the computed total work (the product of the force and the distance through which the body moves expressed in joules based on body weight).

##### Jumping performance

Jumping performance was evaluated by a counter-movement jump (CMJ) and hopping test, both of which are reliable and valid for patients with ATR [28, 32, 36]. Jump height and hopping ratio was measured by the MuscleLab® infrared light beam measurement system. In the CMJ, the patients stood on one leg and they were then asked to quickly bend their knee and perform a vertical jump as high as possible. Three jumps were allowed on each side and the highest jump (cm) was recorded and used in the analysis. The hopping test is a continuous rhythmic jump involving the stretch shortening cycle and it measures the ability of the muscle–tendon complex to utilize its elastic properties. Standing on one leg with their arms at their sides, the patients were instructed to perform 15 jumps at a self-selected speed. The hopping ratio (air time/floor contact time) was the outcome measurement used for analysis.

#### Clinical measurements

##### Ultrasound measurement

Achilles tendon length was measured bilaterally using extended field of view ultrasonography (Logiq E BT09 Ultrasound; GE Healthcare Sweden AB). This measurement method has been reported to have excellent test–retest reliability (ICC = 0.898–0.97) and validity (ICC = 0.895) when the length of a healthy Achilles tendon is compared between cadaveric measurements and ultrasound images [38, 39]. The method has however, not yet been validated for postoperative measurements. The distance between the calcaneal osteotendinous junction (OTJ) and the gastrocnemius musculotendinous junction (MTJ) was measured using a wideband array linear probe (4C-RS, 5.0–13.0 MHz). The B-mode at 10 MHz and a depth of 3 cm were used to record the images. Three images of each Achilles tendon were recorded and measured in centimeters with a measurement accuracy of one decimal. The mean value was used for data analysis and results are presented in centimeters.

##### Achilles tendon resting angle

The Achilles tendon resting angle (ATRA) has previously been reported to have excellent test–retest reliability [5]. With the patient in a prone position and the knee passively flexed at 90 degrees, a goniometer with one-degree increments was placed with one arm along the shaft of the fibula aligned with the center of the fibula head and the other arm with the head of the fifth metatarsal. The angle between the arms was used for analysis.

##### Dorsiflexion range of motion and calf circumference

Dorsiflexion of the ankle joint was measured with a goniometer when standing, with both a straight and a bent knee [26]. The calf circumference was measured at the largest area of the calf muscle with a standard tape and measured in 1 mm increments [5]. Repeated measurements were taken until the same value was found for successive measurements.

### Statistical analysis

Statistical analyses were performed using IBM SPSS Statistics for Mac, Version 24. Descriptive data were presented as mean (standard deviation) and median (range). The outcome data were presented descriptively as median (interquartile range, IQR) and range. Wilcoxon’s signed-rank test was used for comparisons of the injured and the healthy side in the same subject. Effect size was calculated using Cohen’s d and interpreted using the criteria of 0.2- < 0.50 = small, 0.50- < 0.80 = medium and 0.80 or greater = large effect [7]. The level of significance was set at p ≤ 0.05. The limb symmetry index (LSI) was defined as the ratio between the injured and the healthy side, expressed as a percentage (injured/healthy × 100). The LSI represents the clinical relevance and comparisons of muscle function between groups are therefore calculated from the LSI. The recovery of acceptable muscle function was defined as an LSI ≥ 80% since this definition previously been used for patients with CATR [29].

## Results

### Demographics

A total of 22 patients with CATR (14 males) were included in the study (Table 1).

**Table 1:** Patient demographics

	**Mean (SD)** **Median (range)**
**Age**	61 (15)
	67 (28; 82)
	*n* = 22
**Patient gender**	*n* = 22
Male	14 (63.6%)
Female	8 (36.4%)
**Weight (kg)**	85 (15)
	81 (60; 114)
	*n* = 21
**Height (cm)**	173 (9)
	171 (157; 190)
	*n* = 21
**BMI (kg/m2)**	28 (4)
	28 (22; 37)
	*n* = 21

*BMI* Body Mass Index, *CATR* Chronic Achilles Tendon Rupture, *N/A* not available

### Outcomes

The patients reported a mean (SD) ATRS of 62 (26) and mean (SD) PAS 3.5 (1.1). The mean (SD) score of the FAOS subscales were 79 (22) on Symptoms and stiffness, 86 (22) on Pain, 87 (19) on Function in daily living, 62 (31) on Sport and recreation and 66 (29) on Quality of life (Table 2).

**Table 2:** Patient-reported outcomes

	**Mean (SD)** **Median (range)**
**ATRS**	62 (26)
	67 (18; 95)
	*n* = 22
**PAS**	3.5 (1.1)
	3.0 (1.0; 6.0)
	*n* = 21
Level 1	4.5%
Level 2	4.5%
Level 3	45.5%
Level 4	22.7%
Level 5	13.6%
Level 6	4.5%
**FAOS** _***Symptoms and stiffness***_	79 (22)
	89 (25; 96)
	*n* = 22
**FAOS** _**Pain**_	86 (22)
	96 (14; 100)
	*n* = 22
**FAOS** _***Function in daily living***_	87 (19)
	94 (31; 100)
	*n* = 22
**FAOS** _***Sport and Recreation***_	62 (31)
	65 (5; 100)
	*n* = 22
**FAOS** _***Quality of Life***_	66 (29)
	72 (0; 100)
	*n* = 22

*AATR* Acute Achilles Tendon Rupture, *CATR* Chronic Achilles Tendon Rupture, *ATRS* Achilles tendon total rupture score, *PAS* Physical activity scale, *FAOS* Foot and ankle outcome score, *N/A* not available, * small effect, ** medium effect, *** large effect

The patients performed less well in the heel-rise test on the injured side compared with the healthy side (Table 3), with a median (IQR) of 20 (10) vs 24 (12) in heel-rise repetitions (*p* = 0.004), 8 (7) vs 10 (8) cm in heel-rise height (*p* < 0.001), 872 (1740) vs 1590 (2145) joule in total heel-rise work (*p* = 0.001) and 0.37 vs 0.48 in hopping ratio (*p* = 0.005). At group level, an acceptable recovery of muscle function was reached in all tests except the heel-rise height and heel-rise total work. There was no difference between injured and healthy side in terms of CMJ and the effect sizes of side-differences in muscle function ranged from small to medium (0.16–0.63).

**Table 3:** Muscle function in patients with CATR

	**Injured side**	**Healthy side**	**LSI**	**Proportion of patients with acceptable function**	**Difference between injured and healthy side**	
	**Median (IQR)** **Min;max**	**Median (IQR)** **Min;max**	**Median (IQR)** **Min;max**	**%**	**Effect size**	***P*-value**
**Heel-rise repetitions**	20 (10)	24 (12)	84 (23)	55	0.46*	**0.004**
	2; 49	17; 55	12; 133			
	*n* = 20	*n* = 20				
**Heel-rise height (cm)**	8 (7)	10 (8)	79 (21)	45	0.62**	** < 0.001**
	3; 15	7; 17	36; 99			
	*n* = 20	*n* = 20				
**Heel-rise total work** **(joule)**	872 (1740)	1590 (2145)	68 (29)	35	0.59**	** < 0.001**
	49; 3438	737; 4143	5; 138			
	*n* = 20	*n* = 20				
**CMJ (cm)**	5 (7)	5 (10)	98 (36)	56	0.16	0.365
	2; 20	3; 17	65; 204			
	*n* = 16	*n* = 16				
**Hopping ratio**	0.37 (0)	0.48 (0)	90 (19)	70	0.63**	**0.005**
	0.30; 0.53	0.31; 0.62	66; 97			
	*n* = 10	*n* = 10				

*CATR* Chronic Achilles Tendon Rupture, *CMJ* Counter Movement Jump, *IQR* Interquartile range, *Limb symmetry index* = injured/healthy side × 100, * small effect, ** medium effect, *** large effect

Patients with CATR exhibited an elongation of the injured Achilles tendon (Table 4) with median (IQR) ATRA of 55° (3) compared to 50° (9) (*p* < 0.001) and median of 22.4 (2.9) cm compared with 20.5 (2.0) cm measured by ultrasound (*p* = 0.06). Calf circumference was smaller on the injured side with a median (IQR) of 37 (4) compared to 38 (4) cm (*p* = 001). There were no side-to-side differences in passive range of motion in dorsiflexion with either a straight or a flexed knee. The effect sizes of side-differences in the clinical measurements ranged from small to medium (0.12–0.54).

**Table 4:** Clinical measurements in patients with CATR

	**Injured side**	**Healthy side**	**Difference between injured and healthy side**	
	**Median (IQR)** **Min;max**	**Median (IQR)** **Min;max**	**Effect size**	***P*-value**
**ATRA (degrees)**	55 (3)	50 (9)	0.54**	** < 0.001**
	50; 76	40; 69		
	*n* = 21	*n* = 21		
**Tendon length (cm)**	22.4 (2.9)	20.5 (2.0)	0.48*	**0.006**
	17.9; 26.1	17.0; 26.1		
	*n* = 16	*n* = 16		
**Dorsiflexion extended knee (degrees)**	32 (12)	30 (12)	0.17	0.279
	17; 40	21; 41		
	*n* = 20	*n* = 20		
**Dorsiflexion flexed knee (degrees)**	32 (14)	35 (17)	0.12	0.448
	18; 48	17; 44		
	*n* = 20	*n* = 20		
**Calf circumference (cm)**	37 (4)	38 (4)	0.52**	**0.001**
	30; 47	33; 46		
	*n* = 21	*n* = 21		

*CATR* Chronic Achilles Tendon Rupture, *ATRA* Achilles tendon resting angle, * small effect, ** medium effect, *** large effect

## Discussion

The aim of the present study was to evaluate the one-year postoperative outcomes in patients with CATR. The results from patient-reported outcomes used in this study suggest that patients with CATR perceive persistent limitations in foot and ankle function one year after surgery. Patients with CATR had recovered acceptable heel-rise capacity at group-level in terms of repetitions, but not height (LSI = 79%) and work (LSI = 68%). Jumping performance had reached acceptable recovery at group level. In addition, the injured Achilles tendon in patients with CATR had elongated, and the injured side had a smaller calf circumference than the healthy side one year after the surgical repair. The results indicate that patients with CATR have persistent limitations in several aspects of function one year after surgery.

The large variation in treatment outcomes between patients with CATR in the present study indicate that some patients appear to recover well, whereas others are more affected one year after surgery. This is not unique to CATR, as similar patterns are reported after AATR and Achilles tendon re-ruptures (ATRR) [32, 42]. Treatment strategies for AATR and ATRR are being continuously studied and improved, but the fact that treatment is delayed in up to 25% of all acute Achilles ruptures is still a matter of great concern. A previous Swedish study that retrospectively examining error events and complaints related to delayed diagnosis and treatment of ATR reported to The Health and Social Care Inspectorate showed that most errors occurred in the primary health care and in patients older than 60 years [9]. The ATR was commonly misdiagnosed, often in the absence of documentation of an adequate physical examination, and out of 46 patient cases, 31% were characterized by non-specific injury events [9]. It should therefore be clarified that ATR also can occur in older patients and that, despite an atypical patient history, the injury should always be considered and excluded through a physical examination [9].

The patients included in the present study reported functional limitations related to their injury and none of them reported a 100% recovery as measured by the ATRS. Patients in the present study scored lower on the ATRS (median 67, range 18 to 95) compared with a previous study by Nilsson Helander et al. [29] of patients treated with free flap from the gastrocnemius aponeurosis for CATR or ATRR (median 83, range 24 to 100). However, in the study by Nilsson Helander et al. the patients were evaluated a median (range) of 29 (12–117) months after surgical treatment, compared with 12 months in the present study and had a considerably lower mean (SD) age of 46 (10.4).

The greatest patient-reported limitations were related to activities including running and jumping. Older age could be argued as a reason for lower scores in this patient group, however, patient age has previously been shown not to be a risk factor for inferior results in the ATRS after AATR, which indicates that the differences rather are associated with functional deficits and possibly also a negative experience of delayed treatment and, in some cases, misdiagnosis [43]. Patients with ATRR, who also have suffered a complication to the initial injury, have previously been reported to have poorer patient-reported outcomes than patients with an AATR, despite a similar recovery of muscle function [42].

At group level, an acceptable recovery level was not reached in terms of heel-rise total work, which was greatly influenced by impairments in mean heel-rise height in both groups. In the study by Nilsson Helander et al. [29], a larger proportion of patients had recovered acceptable heel-rise height and hopping ratio, but fewer had recovered heel-rise repetitions compared with patients with CATR included in the present study. The same surgical method was used as for the majority of patients with CATR in the present study, but there were individual differences in rehabilitation.

It is possible that patients with CATR suffer greater atrophy and strength loss in the lower leg muscles compared with patients receiving early treatment, due to the delay in the initial diagnosis and treatment. Speculatively, this could lead to a longer recovery process and that full potential of recovery might not be reached at one year postoperatively. Also, differences in preoperative tendon status, surgical methods and post-operative rehabilitation protocols between patients with CATR and AATR may also play a role. Delayed treatment demands a larger surgical procedure and incision to be able to bridge the tendon gap between the retracted and frayed tendon ends. The patients in the present study did not follow a standardized rehabilitation protocol, but rehabilitation after CATR is in general similar to after an AATR, but with a slower progression. They were not allowed to fully load until six weeks postoperatively, however, early weight-bearing in combination with accelerated rehabilitation is associated with positive effects on the healing process and better patient outcomes after AATR compared to traditional immobilization [15]. Accelerated rehabilitation is now more commonly used also in patients with CATR, but not at the time when the data in this study was conducted.

The patients with CATR in the present study exhibited an elongation of the injured tendon compared with the healthy side. Degree of tendon length have previously been reported to affect the outcomes after AATR; less tendon elongation measured by radiographic markers correlates with a more successful outcome, whereas an increased elongation measured by ATRA positively relates to less total work on the heel-rise test after AATR [19, 44]. Only 45% of the patients had recovered acceptable heel-rise height and acceptable recovery of heel-rise total work was reached by 35%. The deficits in heel-rise height in patients treated for CATR might be related to tendon elongation, degenerative changes related to age and/or an injury left untreated for longer than four weeks [39, 41]. Older age at the time of injury and greater intraoperative ATRA (i.e. tightness of repair) has previously been reported to predict inferior heel-rise height one year after AATR [6, 31, 43].

The primary limitations of this study are the small number of patients and the large age age-span between patients. The quality of rehabilitation and patient compliance in the patients with CATR are unknown. It should be emphasized that only 50% of the patients with CATR performed the jump tests and those who chose to abstain from the jump tests did so because of back or lower extremity conditions not related to the ATR. Known weaknesses of case series as a study design is the high risk of selection bias and low internal validity, which affects the robustness and generalizability of the results. Statistical differences between the injured and the healthy side were included to aid in interpretation of the results, but should be interpreted with caution since the case series is a descriptive observational study. The strengths of the study are that it is one of few studies using a comprehensive battery including several validated tests for patient-reported, functional, and tendon property outcomes in patients with CATR, the majority treated with the same surgical technique. Future research is needed to determine whether outcomes after surgical treatment and rehabilitation for CATR are successful or can be further improved.

## Conclusions

Patients with CATR reported persisting limitations in subjective foot and ankle function one year after surgery. The greatest limitations were related to running and jumping. Acceptable heel-rise capacity and hopping ratio were not recovered and there was significant elongation of the repaired tendon compared with the healthy side.

## Data Availability

The datasets used and/or analyzed in the current study are available from the corresponding author on reasonable request.

## Abbreviations

AATR: Acute achilles tendon rupture
ATRA: Achilles tendon resting angle
ATRR: Achilles tendon re-rupture
ATRS: Total achilles tendon rupture score
BMI: Body mass index
CATR: Chronic achilles tendon rupture
CMJ: Counter movement jump
FAOS: Foot and ankle outcome score
N/A: Not applicable
PAS: Physical activity scale

## References

[1] Arshad Z, Lau EJS, Leow SH, Bhatia M (2021). Management of chronic Achilles ruptures: a scoping review. Int Orthop.

[2] Ballas M, Tytko J, Mannarino F (1998). Commonly missed orthopedic problems. Am Fam Physician.

[3] Becher C, Donner S, Brucker J, Daniilidis K, Thermann H (2018). Outcome after operative treatment for chronic versus acute Achilles tendon rupture - A comparative analysis. Foot Ankle Surg.

[4] Bussewitz BW (2017). Repair of neglected Achilles rupture. Clin Podiatr Med Surg.

[5] Carmont MR, Silbernagel KG, Mathy A, Mulji Y, Karlsson J, Maffulli N (2013). Reliability of Achilles tendon resting angle and calf circumference measurement techniques. Foot Ankle Surg.

[6] Carmont MR, Zellers JA, Brorsson A, Nilsson-Helander K, Karlsson J, GrävareSilbernagel K (2020). Age and Tightness of Repair Are Predictors of Heel-Rise Height After Achilles Tendon Rupture. Orthop J Sports Med.

[7] Cohen J (1988). Statistical power analysis for the behavioural sciences.

[8] Čretnik A, KošKir R, Kosanović M (2010). Incidence and outcome of operatively treated Achilles tendon rupture in the elderly. Foot Ankle Int.

[9] Fridén T, Movin T, Andrén-Sandberg Å (2017). Missad diagnos av hälseneruptur vanligast hos äldre patienter. Lakartidningen.

[10] Ganestam A, Kallemose T, Troelsen A, Barfod KW (2016). Increasing incidence of acute Achilles tendon rupture and a noticeable decline in surgical treatment from 1994 to 2013. A nationwide registry study of 33,160 patients. Knee Surgery, Sports Traumatology, Arthroscopy.

[11] Grimby G (1988). Physical activity and effects of muscle training in the elderly. Ann Clin Res.

[12] Gross CE, Nunley JA (2017). Treatment of neglected Achilles tendon ruptures with interpositional allograft. Foot Ankle Clin.

[13] Hadi M, Young J, Cooper L, Costa M, Maffulli N (2013). Surgical management of chronic ruptures of the Achilles tendon remains unclear: a systematic review of the management options. Br Med Bull.

[14] Houshian S, Tscherning T, Riegels-Nielsen P (1998). The epidemiology of Achilles tendon rupture in a Danish county. Injury.

[15] Huang J, Wang C, Ma X, Wang X, Zhang C, Chen L (2015). Rehabilitation regimen after surgical treatment of acute Achilles tendon ruptures: a systematic review with meta-analysis. Am J Sports Med.

[16] Huttunen TT, Kannus P, Rolf C, Felländer-Tsai L, Mattila VM (2014). Acute Achilles tendon ruptures: incidence of injury and surgery in Sweden between 2001 and 2012. Am J Sports Med.

[17] Jozsa L, Kvist M, Balint B, Reffy A, Jarvinen M, Lehto M, Barzo M (1989). The role of recreational sport activity in Achilles tendon rupture: a clinical, pathoanatomical, and sociological study of 292 cases. Am J Sports Med.

[18] Järvinen TA, Kannus P, Maffulli N, Khan KM (2005). Achilles tendon disorders: etiology and epidemiology. Foot Ankle Clin.

[19] Kangas J, Pajala A, Ohtonen P, Leppilahti J (2007). Achilles tendon elongation after rupture repair: a randomized comparison of 2 postoperative regimens. Am J Sports Med.

[20] Kauwe M (2017). Acute Achilles tendon rupture: clinical evaluation, conservative management, and early active rehabilitation. Clin Podiatr Med Surg.

[21] Kraeutler MJ, Purcell JM, Hunt KJ (2017). Chronic Achilles tendon ruptures. Foot Ankle Int.

[22] Levi N (1997). The incidence of Achilles tendon rupture in Copenhagen. Injury.

[23] Maffulli N (1998). The clinical diagnosis of subcutaneous tear of the Achilles tendon. Am J Sports Med.

[24] Maffulli N (1999). Rupture of the Achilles tendon. J Bone Joint Surg Am.

[25] Maffulli N, Via AG, Oliva F (2017). Chronic Achilles Tendon Rupture. Open Orthop J.

[26] Munteanu SE, Strawhorn AB, Landorf KB, Bird AR, Murley GS (2009). A weightbearing technique for the measurement of ankle joint dorsiflexion with the knee extended is reliable. J Sci Med Sport.

[27] Naldo J, Agnew P, Brucato M, Dayton P, Shane A (2021). ACFAS Clinical Consensus Statement: Acute Achilles Tendon Pathology. J Foot Ankle Surg.

[28] Nilsson-Helander K, GrävareSilbernagel K, Thomee R, Faxen E, Olsson N, Eriksson BI, Karlsson J (2010). Acute Achilles tendon rupture: a randomized, controlled study comparing surgical and nonsurgical treatments using validated outcome measures. Am J Sports Med.

[29] Nilsson-Helander K, Sward L, Silbernagel KG, Thomee R, Eriksson BI, Karlsson J (2008). A new surgical method to treat chronic ruptures and reruptures of the Achilles tendon. Knee Surg Sports Traumatol Arthrosc.

[30] Nilsson-Helander K, Thomeé R, Grävare-Silbernagel K, Thomeé P, Faxén E, Eriksson BI, Karlsson J (2007). The Achilles tendon total rupture score (ATRS) development and validation. Am J Sports Med.

[31] Olsson N, Petzold M, Brorsson A, Karlsson J, Eriksson BI, GrävareSilbernagel K (2014). Predictors of clinical outcome after acute Achilles tendon ruptures. Am J Sports Med.

[32] Olsson N, Silbernagel KG, Eriksson BI, Sansone M, Brorsson A, Nilsson-Helander K, Karlsson J (2013). Stable surgical repair with accelerated rehabilitation versus nonsurgical treatment for acute Achilles tendon ruptures: a randomized controlled study. Am J Sports Med.

[33] Reiman M, Burgi C, Strube E, Prue K, Ray K, Elliott A, Goode A (2014). The utility of clinical measures for the diagnosis of Achilles tendon injuries: a systematic review with meta-analysis. J Athl Train.

[34] Roos EM, Brandsson S, Karlsson J (2001). Validation of the foot and ankle outcome score for ankle ligament reconstruction. Foot Ankle Int.

[35] Sheth U, Wasserstein D, Jenkinson R, Moineddin R, Kreder H, Jaglal SB (2017). The epidemiology and trends in management of acute Achilles tendon ruptures in Ontario, Canada: a population-based study of 27 607 patients. Bone Joint J.

[36] Silbernagel KG, Gustavsson A, Thomeé R, Karlsson J (2006). Evaluation of lower leg function in patients with Achilles tendinopathy. Knee Surg Sports Traumatol Arthrosc.

[37] Silbernagel KG, Nilsson-Helander K, Thomeé R, Eriksson BI, Karlsson J (2010). A new measurement of heel-rise endurance with the ability to detect functional deficits in patients with Achilles tendon rupture. Knee Surg Sports Traumatol Arthrosc.

[38] Silbernagel KG, Shelley K, Powell S, Varrecchia S (2016). Extended field of view ultrasound imaging to evaluate Achilles tendon length and thickness: a reliability and validity study. Muscles Ligaments Tendons J.

[39] Silbernagel KG, Steele R, Manal K (2012). Deficits in heel-rise height and achilles tendon elongation occur in patients recovering from an Achilles tendon rupture. Am J Sports Med.

[40] Suchak AA, Bostick G, Reid D, Blitz S, Jomha N (2005). The incidence of Achilles tendon ruptures in Edmonton. Canada Foot Ankle Int.

[41] Wang JH-C (2006). Mechanobiology of tendon. J Biomech.

[42] Westin O, Nilsson Helander K, Grävare Silbernagel K, Samuelsson K, Brorsson A, Karlsson J (2018) Patients with an Achilles tendon re-rupture have long-term functional deficits in function and worse patient-reported outcome than primary ruptures. Knee Surg. Sports Traumatol. Arthrosc 26(10):3063–3072 10.1007/s00167-018-4952-0 PMC6154022 29691618

[43] Westin O, Svedman S, Senorski EH, Svantesson E, Nilsson-Helander K, Karlsson J, Ackerman P, Samuelsson K (2018). Older Age Predicts Worse Function 1 Year After an Acute Achilles Tendon Rupture: A Prognostic Multicenter Study on 391 Patients. Orthop J Sports Med.

[44] Zellers JA, Carmont MR, Silbernagel KG (2018). Achilles Tendon Resting Angle Relates to Tendon Length and Function. Foot Ankle Int.

